# A case report of olmesartan‐associated sprue‐like enteropathy: Diagnosis and healing confirmed by capsule endoscopy

**DOI:** 10.1002/deo2.142

**Published:** 2022-06-18

**Authors:** Yoshiharu Yamaguchi, Takahiro Miwa, Ryo Murakami, Akane Sugimura, Kazuhiro Yamamoto, Tomoya Sugiyama, Yasuhiro Tamura, Shinya Izawa, Masahide Ebi, Yasushi Funaki, Naotaka Ogasawara, Makoto Sasaki, Kunio Kasugai

**Affiliations:** ^1^ Department of Gastroenterology Aichi Medical University School of Medicine Aichi Japan; ^2^ Department of Gastroenterology Tajimi City Hospital Gifu Japan

**Keywords:** atrophy, capsule endoscopy, diarrhea, hypertension, olmesartan

## Abstract

Herein, we describe a case of olmesartan‐related sprue‐like enteropathy in which improvement in villous atrophy was confirmed by small‐bowel capsule endoscopy (CE). We successfully treated a 66‐year‐old man with a chief complaint of loose diarrhea. The patient had persistent watery diarrhea 10 times a day and experienced a weight loss of 9 kg in 3 months. An abdominal computed tomography scan showed fluid retention in the small intestine. Blood test results revealed no inflammatory reaction. Esophagogastroduodenoscopy detected villous atrophy in the stomach and duodenum. Moreover, small‐bowel CE showed villous atrophy in about two‐thirds of the small intestine. Based on other examinations, hyperthyroidism, intestinal tuberculosis, intestinal amyloidosis, and intestinal malignant lymphoma were ruled out. Therefore, the patient was suspected of having an olmesartan‐related sprue‐like disease. Early after discontinuation of medication, diarrhea symptoms improved, and a repeat CE indicated improvements in small intestinal villous atrophy. Since the patient had been administered olmesartan for a long time and CE showed villous atrophy throughout the small bowel, we suspected him of having the olmesartan‐associated sprue‐like disease. The findings of gastric mucosa atrophy on esophagogastroduodenoscopy may lead to an early diagnosis of this disease. Olmesartan‐related sprue‐like enteropathy should be considered as a differential diagnosis in patients with chronic severe watery diarrhea.

## INTRODUCTION

Olmesartan‐related sprue‐like enteropathy is a malabsorption syndrome characterized by chronic diarrhea and weight loss that develops after olmesartan initiation. The symptom onset often occurs from several months to years after starting this medication.^1^ The number of cases of olmesartan‐related sprue‐like enteropathy is increasingly reported in Japan.^2^, ^3^, ^4^, ^5^ Herein, we describe a case of the olmesartan‐related sprue‐like disease in which improvement in villous atrophy was confirmed by small bowel capsule endoscopy (CE). In this case, gastric mucosal atrophy could be visualized by esophagogastroduodenoscopy.

## CASE REPORT

A 66‐year‐old man was referred to our hospital by an outpatient clinic with a 60‐day history of watery diarrhea. His family medical history was unremarkable. His bowel habits comprised persistent watery diarrhea 10 times per day. The patient had a history of hypertension, hyperlipidemia, and 6‐year treatment with nifedipine (20 mg/day), olmesartan (20 mg/day), and atorvastatin (5 mg/day) prescribed at another hospital.

**FIGURE 1:**
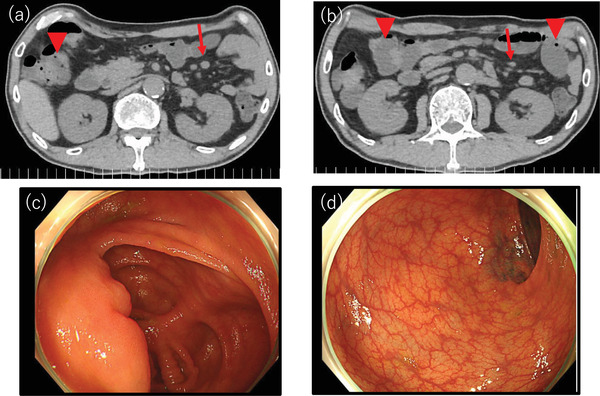
Computed tomography scan of the abdomen: an accumulation of fluids in the small bowel (arrowhead) is noticeable. (a,b) Intra‐abdominal lymphadenopathy with a maximum diameter of approximately 10 mm is observed (arrow). (c,d) Colonoscopy: no obvious anomalies are noted

**FIGURE 2:**
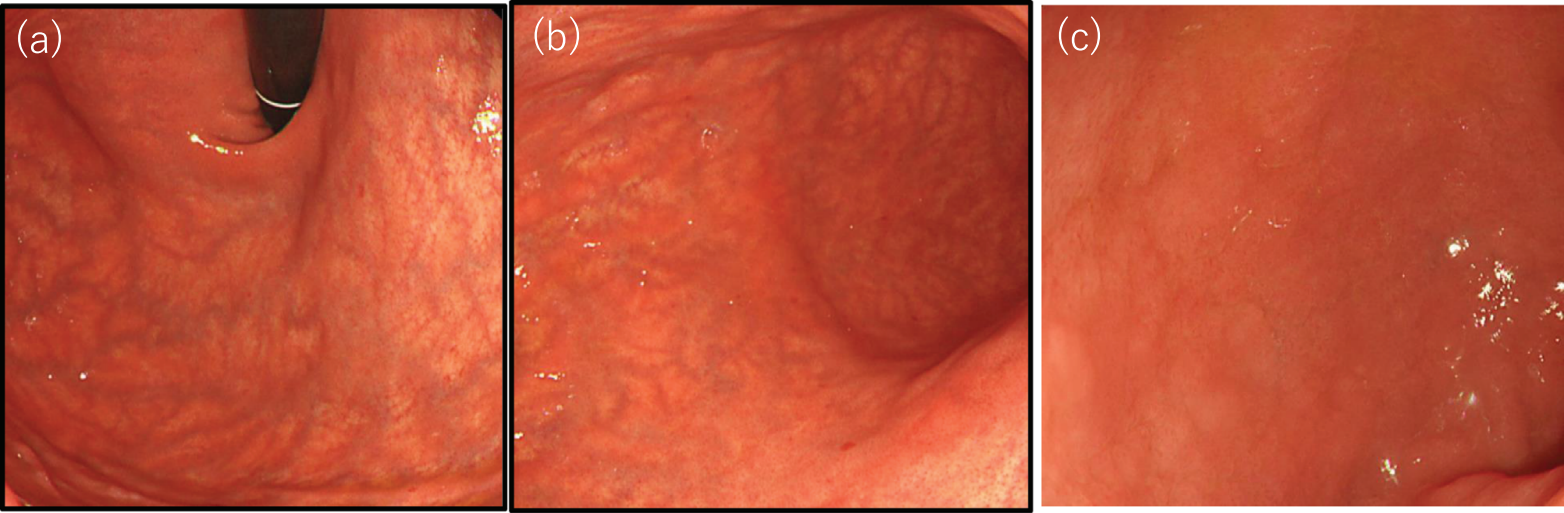
Esophagogastroduodenoscopy: atrophy of the (a,b) gastric mucosa and (c) villous atrophy and scalloping of the duodenum are observed

**FIGURE 3:**
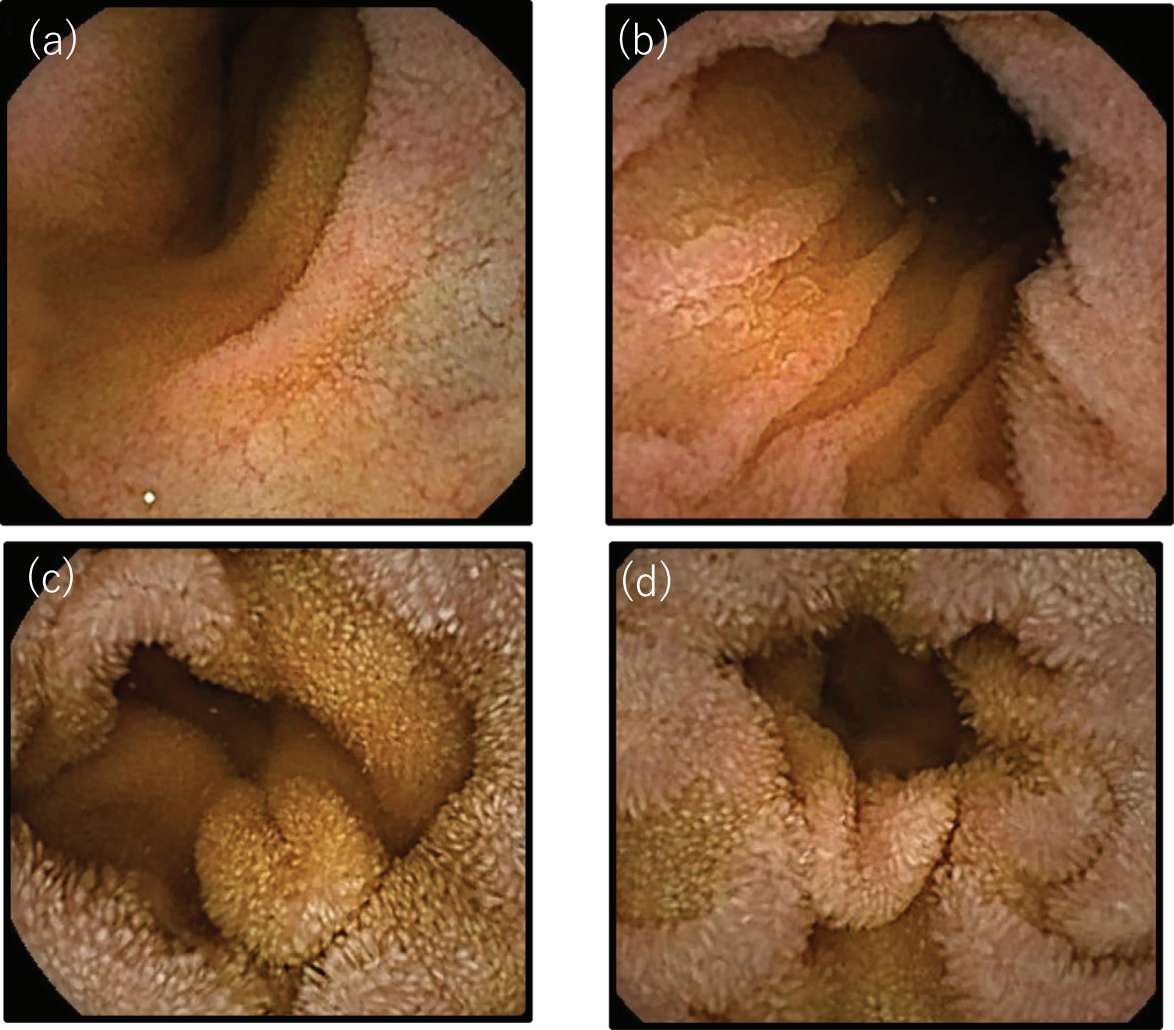
(a,b) Capsule endoscopic findings of the small intestine before discontinuation of olmesartan. Diffusely flattened mucosal surface is observed. The villi are lowered in height. (c,d) Capsule endoscopy of the small intestine 1.5 months after discontinuation of olmesartan: the villi of the small bowel are elevated and a finger‐like villus structure is observed. The findings show improvement in villous atrophy

Physical examination upon his admission was unremarkable. Laboratory test results showed hemoglobin, 14.9 g/dl (normal reference values, 13.9–16 g/dl). These results also revealed white blood cell count, 6.1 × 10^3^/μl (normal values, 5–8 × 10^3^/μl): neutrophil count (4624/μl), lymphocyte count (964/μl), and monocyte count (470/μl). Platelet count, 265 × 10^3^ /μl (normal values, 138–309 × 10^3^ /μl); C‐reactive protein level, 0.07 mg/dl (normal values, <0.3 mg/dl); aspartate aminotransferase level, 20 U/L (normal values, 13 ± 33 U/L); and alanine aminotransferase level, 22 U/L (normal values 6–30 U/L) were also detected. In addition, these laboratory test results indicated total bilirubin level, 0.54 mg/dl (normal values, 0.3–1.2 mg/dl); alkaline phosphatase level, 205 U/L (normal values, 115 ± 359 U/L); and γ‐glutamyl transferase level, 253 U/L (normal values, 10–47 U/L). Abdomen‐pelvis computed tomography scan revealed fluid retention in the small intestine and intra‐abdominal lymphadenopathy with a maximum diameter of approximately 10 mm (Figure 1a,b). Colonoscopy on admission showed no apparent abnormalities (Figure 1c,d). Additionally, esophagogastroduodenoscopy revealed gastric mucosal atrophy with a regular arrangement of collecting venules (Figure 2a,b) and duodenal villous atrophy (Figure 2c). Small bowel CE showed villous atrophy in approximately two‐thirds of the small bowel (Figure 3a,b). Additional testing for *Helicobacter pylori*, anti‐parietal cell, and anti‐intrinsic factor antibodies was negative. Simultaneously, the laboratory test showed negative results for serum amyloid (3.7 μg/ml), *Mycobacterium tuberculosis*‐specific Interferon‐γ, soluble interleukin 2 receptor (621 U/ml), and thyroid markers (free triiodothyronine: 1.94 pg/ml, free thyroxine: 1.24 ng/dl, and thyroid‐stimulating hormone: 1.41 μIU/ml). The initial provisional diagnosis was olmesartan‐associated sprue‐like enteropathy. Therefore, the patient discontinued olmesartan administration. Thereafter, the frequency of diarrhea gradually improved to about three times per day. The patient was discharged 1 week after admission. Ten days after discharge from the hospital, he had loose stools once a day. Forty days after discharge, a small bowel CE showed that the villus length in the small intestine had increased and was considered almost normal (Figure 3c,d). Antihypertensive medications were changed at another hospital. Five months after discharge, the patient was found to be free of any clinical symptoms.

## DISCUSSION

According to the World Health Organization report, over one billion people worldwide experience hypertension. It is the leading cause of premature death. Olmesartan has been widely used worldwide, including in Japan, since its approval as an antihypertensive drug by the Food and Drug Administration in 2002.^6^ Olmesartan‐associated sprue‐like enteropathy was proposed by Tapia et al. in 2012 who reported 22 patients with this adverse effect. Symptoms include nausea, vomiting, diarrhea, weight loss, and electrolyte abnormalities common to celiac disease. The mean duration was 3.1 years (range, 0.5–7 years) between drug initiation and the onset of diarrhea. These symptoms have been reported to improve promptly after discontinuation of olmesartan.^1^ In our case, as well, symptoms improved promptly after drug discontinuation. In addition, CE 1.5 months after discontinuation of olmesartan showed rapid improvement in small intestinal mucosal atrophy.

The clinical features of olmesartan‐related sprue‐like enteropathy from previously reported cases in Japan are summarized in Table 1. Our patient is the fifth Japanese patient documented to have olmesartan‐related sprue‐like enteropathy and the first patient documented to have healed atrophic mucosa confirmed by CE. Olmesartan‐associated sprue‐like enteropathy is a disease that extensively affects the intestinal tract. Unlike other endoscopic procedures, small intestinal CE is extremely useful and unique because it allows a detailed examination of the entire small intestinal mucosa, which is difficult to evaluate using routine diagnostic endoscopy. Moreover, the procedure is painless, easy to perform, and repeatable.

**TABLE 1:** Clinical features of patients with olmesartan‐associated sprue‐like enteropathy based on previous case series and reports in Japan

**Reference**	**Sex/age**	**Symptoms**	**Duration of olmesartan use before symptoms**	**Diagnostic modalities**	**Endoscopic findings**	**Length of time from drug cessation to clinical resolution**
Uehara et al.^2^	M/76	Diarrhea	3 years	EGD and CS	Atrophy of the gastric mucosa	Information not provided
Kaneko et al.^3^	M/73	Diarrhea and weight loss	5 years	EGD and CS	Villous atrophy of the duodenum and terminal ileum	3 weeks
Taguchi et al.^4^	F/81	Diarrhea	10 years	CE, EGD, CS, and DBE	Villous atrophy of the small intestine	1 week
Sasaki et al.^5^	F/75	Diarrhea and weight loss	1.5 years	EGD	Villous atrophy of the duodenum	1 week
Present case	M/66	Diarrhea and weight loss	6 years	CE, EGD, and CS	Atrophy of the gastric mucosa and villous atrophy of the small intestine	1 week

Abbreviations: CE, capsule endoscopy; CS, colonoscopy; DBE, double‐balloon endoscopy; EGD, esophagogastroduodenoscopy; F, female; M, male.

The hypothesized mechanisms include the following:

First, the expression of transforming growth factor‐β, an anti‐inflammatory cytokine that plays an important role in maintaining intestinal mucosal immunity, is suppressed by angiotensin (Ang) receptor blockers, increasing the levels of Ang II and resulting in a relatively elevated level of proinflammatory cytokines.^1,^, ^7^ Second, Ang II is inhibited from binding to Ang II Type 1 receptors, which are present almost everywhere in the gastrointestinal tract and are thought to bind to Ang II Type 2 receptors and promote apoptosis, resulting in small intestinal villous atrophy.^8^ However, it has been reported that symptoms disappeared after switching from olmesartan to losartan,^9^ suggesting that there is a specific mechanism for olmesartan. Therefore, further research is required.

Esophagogastroduodenoscopy revealed diffuse mucosal atrophy. Based on blood tests, antibodies of *H. pylori* or autoimmune gastritis such as anti‐parietal cell antibodies and anti‐intrinsic factor antibodies were negative. Therefore, the patient was considered to have olmesartan‐induced gastric mucosal atrophy. This disease also causes pathologically active chronic gastritis.^10^ One of the differential diagnoses of atrophic gastritis should be considered drug‐induced by olmesartan.

Based on our case, olmesartan‐associated sprue‐like enteropathy is often difficult to diagnose and should be considered one of the differential diagnoses of chronic severe watery diarrhea.

## CONFLICT OF INTERESTS

None.

## FUNDING INFORMATION

None.

## ETHICS STATEMENT

All procedures performed in studies involving human participants were in accordance with the ethical standards of the institutional and/or national research committee and with the 1964 Helsinki declaration and its later amendments or comparable ethical standards. Written informed consent was obtained from the participant for publication of this case report.
